# Indel-tolerant read mapping with trinucleotide frequencies using cache-oblivious *kd*-trees

**DOI:** 10.1093/bioinformatics/bts380

**Published:** 2012-09-03

**Authors:** Md Pavel Mahmud, John Wiedenhoeft, Alexander Schliep

**Affiliations:** ^1^ Department of Computer Science; ^2^ BioMaPS Institute for Quantitative Biology, Rutgers University, New Jersey, USA

## Abstract

**Motivation:** Mapping billions of reads from next generation sequencing experiments to reference genomes is a crucial task, which can require hundreds of hours of running time on a single CPU even for the fastest known implementations. Traditional approaches have difficulties dealing with matches of large edit distance, particularly in the presence of frequent or large insertions and deletions (indels). This is a serious obstacle both in determining the spectrum and abundance of genetic variations and in personal genomics.

**Results:** For the first time, we adopt the approximate string matching paradigm of *geometric embedding* to read mapping, thus rephrasing it to nearest neighbor queries in a *q*-gram frequency vector space. Using the *L*_1_ distance between frequency vectors has the benefit of providing lower bounds for an edit distance with affine gap costs. Using a cache-oblivious *kd*-tree, we realize running times, which match the state-of-the-art. Additionally, running time and memory requirements are about constant for read lengths between 100 and 1000 bp. We provide a first proof-of-concept that geometric embedding is a promising paradigm for read mapping and that *L*_1_ distance might serve to detect structural variations. TreQ, our initial implementation of that concept, performs more accurate than many popular read mappers over a wide range of structural variants.

**Availability and implementation:** TreQ will be released under the GNU Public License (GPL), and precomputed genome indices will be provided for download at http://treq.sf.net.

**Contact:** pavelm@cs.rutgers.edu

**Supplementary information:** Supplementary data are available at *Bioinformatics* online.

## 1 INTRODUCTION

The possibility to re-sequence genomes rapidly and cost-efficiently using next generation sequencing (NGS) technologies has provided fascinating insights into the breadth and prevalence of human genetic variation (The 1000 Genomes Project Consortium, 2010; The International HapMap Consortium, 2005), in particular the abundance of structural variants—we will jointly refer to them as insertion and deletions (indels) and not distinguish, for example between novel sequence insertions and duplications. Unfortunately, these structural variants, more exactly short indels, are complicating the first step in the analysis, mapping DNA sequencing reads to reference genomes.

This is surprising, as approximate string matching, the theoretical problem underlying read mapping, is arguably one of the most fundamental problem in bioinformatics and a very well-studied area in data mining; for surveys see Boytsov (2011b); Navarro (2001). Mapping reads from DNA sequencing experiments requires solving approximate string matching problems for billions of short DNA sequences of length 20–500 bp against entire genomes. There have been a multitude of methods proposed—see for example the benchmarks performed by Hach *et al.* (2010)—and the results and optimal choice of method depend strongly on the read length and the maximal edit distance allowed.

Out of the variety of different approaches [see Boytsov (2011a) for a detailed taxonomy] proposed for approximate string matching, current read mappers rely on only three different paradigms (Li and Homer, 2010): seed-and-extend (encompassing hash tables and *q*-gram filtering), prefix/suffix tries (using the Burrows–Wheeler transform) and one approach based on merge sort (Malhis and Jones, 2010). Their computational efficiency depends on the existence of exact matches between the read and the genome.

**Fig. 1.**
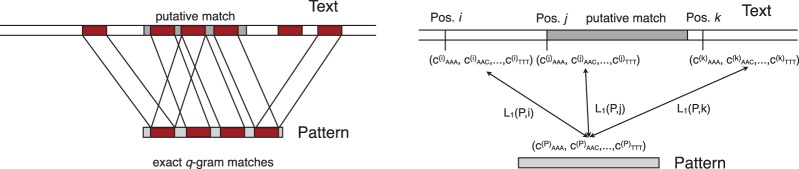
Most approaches to approximate string matching using Ukkonen's *q*-gram lemma rely on the existence of reasonably large *q*-grams which are exact matches between pattern and text. These can be found efficiently with a number of techniques and yield putative hits, which are then evaluated using an alignment algorithm. For each pattern and each putative hit, the number of shared *q*-grams is evaluated *de novo* (left). We map both reads and genome locations to vectors of 2-gram frequencies and identify approximate matches finding nearest neighbors (right). This is accelerated by the use of a spatial index structure, e.g. a *kd*-tree, which is created by recursively partitioning the input space around the median value of a dimension.

Intuitively, there cannot be an approximate match of small edit distance between a read and the genome if not one or several exact matches of length *q* exist. The relationship between the presence of such matching *q*-grams (sequence of length *q*) and the edit distance was revealed in a seminal paper by Ukkonen (1992): a lower bound for the edit distance between two strings is given by the *L*_1_ distance between their count vectors of *q*-grams (for *q*=3 these are the trinucleotide frequency vectors). This provides the basis for a seed-extend strategy of using efficient algorithms for finding one initial exact *q*-gram match, exploring whether additional exact *q*-gram matches support the existence of an approximate match, Figure 1 (left), and then use an efficient alignment algorithm, such as Myers' bit-vector algorithm (Myers, 1999), to verify and assess the quality of the match. Existing methods either implement this idea of *q*-gram filtering (Navarro *et al.*, 2005) directly (Weese *et al.*, 2009), or implicitly rely on it (Li and Durbin, 2009).

The running times depend on the maximal edit distance permitted: smaller maximal edit distance allows to chose larger *q*, thus there will be fewer exact *q*-gram matches and putative approximate matches to explore; indeed their probability decreases exponentially. If we think of patterns being derived from a match in the text through edit operations of technical nature (sequencing errors), or biological nature (genetic variants), the probability of hitting all *q*-grams and thus rendering *q*-gram filtering useless increases with the number of edits (Sutinen and Szpankowski, 1998). Gapped *q*-grams (Burkhardt and Kärkkäinen, 2002), requiring exact matches in a fixed pattern of *q* out of *q*′ > *q* positions, are one way of addressing this. Most popular approaches, however, strictly limit the maximal edit distance and use heuristics to keep running time in check at the potential cost of missing best matches.

In particular, the detection of indels suffers from the limits on edit distance of matches. As our results show, many of the existing methods have problems in mapping 100 bp reads with indels to the reference genome; longer reads improve the situation for some approaches. Consequently, the state-of-the-art in the detection of structural variants is the use of paired-end read libraries and advanced methods for performing downstream analysis after mapping the paired-end read libraries to reference genomes (Chen *et al.*, 2009; Hormozdiari *et al.*, 2009, 2010; Korbel *et al.*, 2009; Lee *et al.*, 2009). Nevertheless, Alkan *et al.* (2011) noted that in particular detection of small, 5–50 bp indels, is a largely open problem, although our analysis reveals that one recent approach, Stampy (Lunter and Goodson, 2011), provides excellent sensitivity. In the detection of such short indels, the deviations from mean insert length are measured, and thus, the sequencing coverage required to arrive at statistical significance is inversely proportional to the indel length. Our results will show that the detection of 1–16 bp indels from single reads is possible using *L*_1_ distance.

We pursue a different strategy from current read mappers, following ideas first proposed for protein sequences (Bugnion *et al.*, 1995) and generally referred to as vector space frequency distance methods (Boytsov, 2011b), embedding strings as *q*-gram frequency vectors. These geometric embeddings have not yet made their way into read mapping, unlike other areas of bioinformatics, for example in the estimation of bacterial species phylogeny through oligonucleotide frequency distances (Takahashi *et al.*, 2009), under the name of *k*-spectra in classifying protein sequences using support vector machines (Leslie *et al.*, 2002) or in alignment-free sequence comparisons (Goke *et al.*, 2012; Liu *et al.*, 2011; Reinert *et al.*, 2009; Wan *et al.*, 2010).

We choose *q* = 3 and consider vectors of all trinucleotide frequencies, by embedding reads of length between 100 and 1000 bp as vectors in ℝ^64^. The problem of finding a minimal edit distance approximate match now becomes the problem of finding a nearest neighbor in a data set of vectors derived from a genome by sliding a window over the genome and mapping the sequence to a frequency vector, Figure 1 (right). Finding (approximate) nearest neighbors, however, has been well studied and a large range of spatial index data structures have been proposed (Berchtold *et al.*, 1996; Bern, 1993; Katayama and Satoh, 1998; Sellis *et al.*, 1987) generally leading to *O*(*n*log*n*) complexity for construction of the spatial index and *O*(log*n*) complexity for nearest neighbor queries, where *n* denotes the number of points in the index. Empirical running times, however, vary widely based on the detailed structure of the problem instance, and thus, algorithm engineering is important for achieving competitive running times.

The vector space frequency distance method introduced in Bugnion *et al.* (1995) was not further pursued except in a small scale study focusing on different ways to map strings to vectors (Ozturk and Ferhatosmanoglu, 2003, 2005). In recent years, researchers in databases, both multi-media and text, investigated indices in high-dimensional spaces (Böhm *et al.*, 2001; Bustos and Navarro, 2009; Houle and Sakuma, 2005; Navarro and Chávez, 2006; Yao *et al.*, 2010), but the small alphabet size of DNA that leads to non-sparse frequency vectors preclude their use here. Boytsov (2011b) implemented and evaluated a range of different approaches in approximate string matching also on DNA datasets which are of small bacterial genome size (3.2 megabasepair). We found that his findings do not translate when the genome size increases by a factor of 1000. For instance the effects of cache or page misses, which motivate cache-oblivious data structures that guarantee minimum number of cache misses irrespective of cache size and memory hierarchy, are simply not observable on small data sets. During the development of the method, we used state-of-the-art *kd*-tree libraries (Mount and Arya, 2010; Muja and Lowe, 2009) but found them to be lacking in performance once the index contained more than a few million points. Indeed, on genome-size problems, the ability to effectively implement data structures in a cache-oblivious manner is more important than computational complexity.

In the following sections, we will show how *L*_1_ distance serves as a lower bound for affine gap costs, introduces our methodology and implementation details and provides detailed analysis on both real and simulated data to show the advantages and drawbacks of geometric embeddings.

## 2 METHODS

We use the usual notation, following (Gusfield, 1997): A finite set of characters ∑ = {*a,b,c,...*} we will call an alphabet and a sequence *s* of characters from ∑ a string. We denote by |*s*| its length, by *s_i_* its *i*-th character, *i* > 0, and by *s*[*i,j*] the continuous sub-string starting at position *i* and ending in position *j*.

We associate strings with vectors by computing the frequencies of all *q*-grams,
(1)
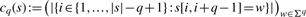

which define a map from ∑* → ℝ^|∑|*^q^*^ through *s* ↦ *c_q_* (*s*). We assume that the *q*-words are in lexicographic order.

Definition 1. *The edit distance* ED(*s,t*) *between two strings s and t is determined by the minimal number of edit operations—substitutions, insertions and deletions—necessary to transform one into another. We can notate the edit operations as rewriting rules, a → ∈ is a deletion, ∈ → a an insertion and a → b a substitution. Here, a,b ∈∑, a ≠ b and ∈ is the empty string. What is usually referred to as* the *edit distance is indeed the Levenshtein distance which assigns unit costs to the three possible operation. This of course generalizes to arbitrary costs c_s_*,* c_i_ and c_d_ for substitutions, insertions and deletions, respectively.*

Similarly we obtain a distance from the *q*-spectrum by considering the *L*_1_ distance of the count vectors in ℝ^|∑|*^q^*^, *L*_1_(*s,t*):=|*c_q_* (*s*)–*c_q_* (*t*)|.

Theorem 1 (Ukkonnen (Ukkonen, 1992)). *For s,t ∈* ∑*^q^*
(2)
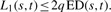


Note that the bound can become arbitrarily bad, for example when *t* is a rotation or transposition of *s*, see Ukkonen (1992).

### 2.1 The *q*-gram lemma revisited

Ukkonen's lemma states that *L*_1_ ≤ 2*q*ED, but this bound is dominated by the mismatches. It is worthwhile to consider the effects of mismatches and indels separately. Consider two strings *S*_1_ and *S*_2_, where *S*_2_ is derived by a single deletion of size *d* from *S*_1_ or by insertion vice versa. Then the *L*_1_ distance comprised two components. First, the number of *q*-grams spanning the gap in *S*_2_ is *q*–1. Second, for *S*_1_, the first character in the deletion accounts for *q* deletions of *q*-grams; however, every consecutive character only accounts for one, as the other *q* – 1 are already accounted for by deleting the left neighbor. Hence, the *L*_1_ distance is bounded by *L*_1_ ≤ 2*q*+*d* – 2. As a single mismatch can affect at most 2*q* number of different *q*-grams, it follows that for *m* mismatches and *g* gaps of size *d_i_*, 1 ≤ *i* ≤ *g*,
(3)
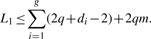

As ∑_*i*=1_^*g*^ *d_i_* + *m* = ED, we obtain
(4)
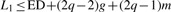

This shows that the number *g* of contiguous gaps (not the total number of gapped positions), provides a sharper bound than the number of mismatched positions. For example, if ED = 4 and *q* = 3, then *L*_1_ ≤ 8 for a single indel of size 4, but *L*_1_ ≤ 24 for four mismatches. Any algorithms based on nearest neighbors under *L*_1_ distance is thus very well suited for mapping reads with large indels.

Apreference for large indels in alignments is biologically more meaningful than alignments with many small indels and generally addressed by using affine gap costs. The above formula naturally implies a scoring scheme for an affine edit distance AED(*s,t*). As
(5)
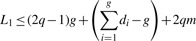

we obtain
(6)
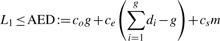

for gap opening cost *c_o_* := 2*q*–1, gap extension cost *c_e_* := 1 and substitution cost *c_s_* := 2*q*.

For edit operations that affect positions *q* letters apart, it is easy to show that the inequality is sharp, that is *L*_1_ gives the edit distance with affine gap costs. Consequently, finding matches of minimal *L*_1_ prefers matches with fewer indels over matches with frequent substitutions. However, the lower bound can be still arbitrarily bad (see previous section), but the probability of catastrophic failure is small (see the Analysis in Supplementary Materials). Indeed the probability of *L*_1_ distance being zero in the presence of one deletion of length *k* is
(7)
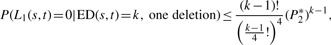

where *P*_2_^*^ is the maximal transition probability in the Markov chain assumed to have generated reads and genome. See Supplementary Material for details.

### 2.2 Read mapping with cache-oblivious *kd*-trees

Efficient searches for exact or inexact nearest neighbors in high dimensions generally involve creating a tree-like index structure that recursively partitions the space. Their efficiency depends on the quality of the index and the geometric distributions of points. A comparative study (Kibriya and Frank, 2007) showed that in a wide range of practical instances *kd*-trees outperform more advanced methods (Berchtold *et al.*, 1996; Bern, 1993; Katayama and Satoh, 1998; Sellis *et al.*, 1987).

In the index generation, for each sub-tree of the *kd*-tree, a dimension—usually the one with the highest variance—is chosen, and then the set of points under the sub-tree is partitioned using the median value of the chosen dimension as pivot. This process continues recursively and eventually completes in *O*(*dn*log*n*) time for *n d*-dimensional points. During the search, a query point's coordinates are compared with the pivot, and a decision to search the left or right sub-tree is made. If there is no exact match to be found, the search procedure backtracks.

As the index size gets larger, the effect of cache misses becomes very prominent and the running time increases substantially. As a result, over the last decade, many important data structures including *kd*-trees were made cache-oblivious (Agarwal *et al.*, 2003; Frigo *et al.*, 1999). Our cache-oblivious *kd*-tree implementation stores the tree in sequential memory using the van Emde Boas layout (van Emde Boas, 1975; van Emde Boas *et al.*, 1976), which guarantees an optimal number of cache misses.

We have implemented the following modification to the usual *kd*-tree construction. During index generation, we use pre-selected dimensions based on the entropy of the dimensions over the full data set, which makes the index building step *O*(*n*log*n*) instead of finding the dimension with highest variance. The minimal axis-parallel hyper-rectangle containing all the points in the subtree defines a bounding box per subtree. These bounding boxes help to reduce the search space at the cost of increasing the index generation to *O*(*dn*log*n*). In the search step, we compute lower bounds for the *L*_1_ distance between the query point and all the points inside the bounding box of the left and right sub-tree in *O*(*d*) time at every subtree. We proceed with the sub-tree giving the best lower bound and store the other sub-tree for future consideration. If no exact match is found the search process becomes expensive. We bound the number of alternate paths searched per query with the parameter *β*, which bounds the running time per query to *O*(*βd* log*n*).

In the bottom levels, the running time overhead to find lower bounds using the bounding boxes is comparable with directly computing the *L*_1_ distances. Thus, for the last *τ* levels, we do not create bounding boxes, and in the search step, we simply compute the *L*_1_ distances between the query point and the points in the sub-tree (we use fast hardware accelerated *L*_1_ distance computation). This also decreases memory requirement to store the tree by 2*^τ^* times.

#### 2.2.1 Verification by Myers' Bitvector algorithm

Using Myers' bit-vector algorithm (Myers, 1999), we compute the edit distance and the exact location for each read based on putative locations identified as nearest neighbors, adding a slack of 14 bp on each boundary. The best position is reported, breaking ties arbitrarily.

#### 2.2.2 Parameter choices

Parameter *β*, the maximum number of different paths explored in the search, controls the running time and sensitivity of TreQ. However, this does not restrict the maximal edit distance of matches in contrast to trie-based methods which avoid exponential blow-up with such restrictions. A second parameter, *τ* influences the memory footprint and running times, as the lowest *τ* levels of the cache-oblivious *kd*-tree are not stored and rather direct *L*_1_ distance computations are performed. To further reduce memory requirements, the genomic window is shifted by *g* base pairs to create *d*-dimensional (*d* = 4*^q^*) frequency vectors (final match positions are based on Myers' alignment). A third parameter *α*, determines number of vectors for which we only store the changes from a nearby point as they can be constructed with minimal overhead from their differences in few dimensions from the (2*α*+1)-*th* point which is actually stored (see Supplementary Material for a detailed explanation). In our experiments we have found *g* = 3, *α* = 2, *β* = 3000 and *τ* = 3 to be a good choice for the human genome, and unless otherwise stated these are the default parameter values for TreQ. Note that for a wide range of parameter choices TreQ's accuracy remains effectively the same (cf. Supplementary Figure S2). In contrast, most popular read mappers have large number of parameters, which are specifically tuned for typical datasets and often very difficult to optimize.

## 3 DISCUSSION

**Fig. 2.**
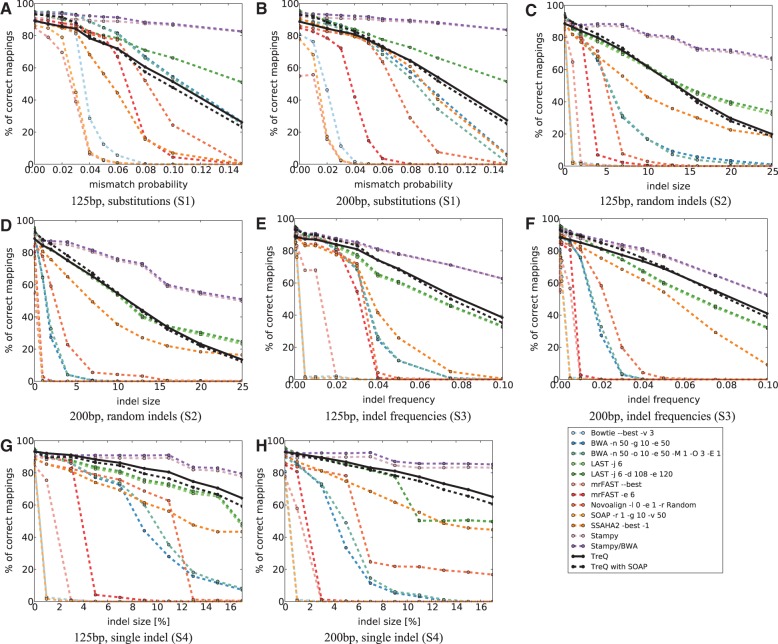
Comparison of popular read mappers with TreQ. Accuracy is defined as the percentage of single best reads that are mapped to the exact genomic location they were drawn from in the simulation. Notice that TreQ outperforms most popular read mappers and is mostly on par with LAST

To evaluate TreQ and compare its performance, we ran a number of read mappers–Bowtie (Langmead *et al.*, 2009), BWA (Li and Durbin, 2009), SOAP2 (Li *et al.*, 2009), mrFAST (Alkan *et al.*, 2009), Novoalign (http://www.novocraft.com), SSAHA2 (Ning *et al.*, 2001), LAST (Frith *et al.*, 2010; Hamada *et al.*, 2011), Stampy (Lunter and Goodson, 2011) and RazerS (Weese *et al.*, 2009)–on simulated and real read datasets (see Supplementary Tables S1 for version numbers). These read mappers were evaluated with their default and, in some cases, customized parameters for allowing maximal permissible edit distance. We also forced them to report one single best hit. For TreQ, parameters *q* = 3, *d* = 64(= 4*^q^*), *g* = 3 and *k* = 200 were fixed throughout the experiments. Currently, quality scores are ignored in the match evaluation phase of TreQ. For simulated data, we define accuracy as the percentage of reads mapped to the actual genomic locations from where they were sampled (Fig. 2). We use human genome HG18 build 36 as the reference for all the experiments.

### 3.1 Simulated data

We simulated four different read data sets, comprising a set of different model parameters, each by sampling 10 000 reads from the human reference genome. Given read length ℓ, we have:

- **S1** 0 to 15ℓ% single-nucleotide substitutions at random positions
- **S2** Indels of size 0–25 at 2ℓ% random locations in the read
- **S3** Indels of size 2 at 0–10ℓ% random locations in the read
- **S4** A single indel of size 0 to 17ℓ% at a random location

On top of that, we simulated sequencing error by estimating and interpolating an Illumina error profile. We estimated a first-order Markov chain of Phred score transitions from 1 million reads of length 101 from a Yoruba African individual (NA18507) for each of those 101 positions. To remove noise and simulate reads of lengths other than 101 bp, we used univariate spline interpolation to estimate the evolution of each entry of the matrix over the read sequence. These functions were then stretched or skewed for other read lengths, and new transition probabilities were derived by evaluating the spline function at the appropriate positions and rescale the rows, so that the matrix becomes stochastic. The resulting Phred score distributions of the reads simulated by iterating the new Markov chains were then verified to correspond to those of real data. As Phred scores correspond to actual error rates (Ewing and Green, 1998), we used them to simulate position-dependent sequencing errors.

Our evaluation shows that Stampy outperforms all other methods in terms of accuracy (Fig. 2). Its running time, however, depends on the type of read. For instance mapping all S1 reads of 500 bp length takes 1 h 35 min, in whereas mapping S2 takes 5 h (Supplementary Table S1). TreQ's running time ranges consistently at about 3.5 h. Stampy's running time also increases with read length, although it is mostly better than TreQ's. LAST performs in about the same accuracy range as TreQ but is not competitive in running time.

In terms of accuracy, geometric embedding outperforms all suffix trie and seed-extend-based read mappers other than Stampy and LAST on almost all instances. The authors of both these programs argue that their accuracy is mainly due to the elaborate downstream analysis they perform after finding candidates. Our results are thus preliminary, as in essence we are comparing statistical alignment models of Stampy and LAST to a simple filter based on Levenshtein distance in our case. BWA can be customized to be competitive on S1 for shorter reads, at the cost of higher running times, but this improvement does not translate to S2–S4. Similarly, LAST outperforms TreQ on S1, but its performance is similar or worse than TreQ's on other conditions. Trie-based read mappers (Bowtie BWA and SOAP2) are very fast but do not perform well in general. Using a hybrid approach—in which SOAP's unmapped reads are mapped by TreQ—increases the accuracy for lower distances at the expense of slightly lower accuracy for higher distances, whereas drastically reducing the running time.

### 3.2 Biological data

Following the evaluation in Hach *et al.* (2010), we compared TreQ to popular read mappers on a set of 1 million randomly selected 101 bp reads from Yoruba African individual (NA18507) (The 1000 Genomes Project Consortium, 2010). The running time and the percentage of reads mapped to the reference human genome HG18 build 36 within 3, 6, 12, and 18 edit distances are reported in Table 1. Times are for a single thread on a single core of a 2.2 GHz AMD Opteron processor.

**Table 1.** One million randomly selected Illumina single end reads mapped to HG18 build 36

Technique	Algorithm	Parameters	Time (h:m)	Mapped percentage ≤ ED			
				≤ 3	≤ 6	≤ 12	≤ 18
	Bowtie	–best	0:04	85.22	–	–	–
		–best -v 3	0:04	86.85	–	–	–
Suffix trie	BWA	default	0:14	87.31	89.35	–	–
		-n 50 -o 10 -e 50 -M 1 -O 3 -E 1	5:53	87.35	90.08	92.39	93.03
	SOAP2	-v 50 -g 10 -r 1	**0:03**	84.87	–	–	–
	mrFAST	–best -e 6	19:50	87.54	90.59	–	–
	Novoalign	-l 0 -e 1 -r Random	0:27	83.68	84.80	85.18	85.19
	SSAHA2	–best -1	45:36+	–	–	–	–
	RazerS	–unique	14:45	66.67	79.41	–	–
		default	1:57	85.73	88.32	90.90	92.15
Seed-extend	Stampy	–bwa-options	0:38	**90.37**	**92.05**	**93.81**	**94.84**
		default	1:32	84.76	87.66	90.23	90.78
		-d108 -e120	1:35	84.85	87.77	90.69	91.69
	LAST	default, LAMA	4:36	68.74	71.12	73.26	73.72
		-d108 -e120, LAMA	4:39	39.26	40.60	41.95	42.42
		*τ* = 1, *β* = 5000, *α* = 0	7:00	87.34	90.12	93.06	94.67
		*τ* = 2, *β* = 4500, *α* = 0	6:28	87.27	90.06	93.01	94.61
		*τ* = 3, *β* = 4000, *α* = 0	6:36	87.22	90.04	93.02	94.62
Geometric embedding	TreQ	*τ* = 1, *β* = 5000, *α* = 2	8:15	87.32	90.11	93.04	94.66
		*τ* = 2, *β* = 4000, *α* = 2	7:44	87.16	89.93	92.87	94.50
		*τ* = 3, *β* = 3000, *α* = 2	8:50	86.90	89.69	92.66	94.30
Hybrid	SOAP2 + TreQ	*τ* = 3, *β* = 3000, *α* = 2	2:06	87.89	90.50	93.26	94.83

The percentages of reads mapped within a fixed edit distance (ED) by various read mappers are reported. As expected, trie-based read mappers are very fast but mostly fail to map reads with higher errors. BWA with customized parameters performs well but with significantly increased running time. Seed-extend-based methods have varied outcomes; mrFAST, RazerS and SSAHA2 take significantly more running time than others, Novoalign is comparably fast but fails to map reads with higher edit distances, whereas LAST (without LAMA option) and Stampy map almost similar amount of reads as TreQ. In contrast to most read mappers, TreQ is not restricted to few mismatches, small indels or few number of indels, and maps either an almost similar percentage of reads or more with various different parameter settings. TreQ's running time is significantly lower than mrFAST, RazerS and SSAHA2 and comparable with customized BWA; we stopped SSAHA2 after it did not finish running in 45 h. Additionally, Hybrid TreQ/SOAP outperforms most read mappers, whereas significantly reducing the required running time. Note that Bowtie only allows mismatches and is restricted to at most 3. All running times are based on running the read mappers single threaded on a single core of a 2.2 GHz AMD Opteron processor.

We have found suffix-trie-based read mappers, implemented using Burrows–Wheeler transform, to be very fast on the real dataset but, unsurprisingly, limited in their ability to map reads with higher edit distances. Seed-extend-based techniques in contrast usually map more reads at large edit distances but require more CPU time. Except LAST, Stampy and TreQ, none of the other read mappers that we have evaluated successfully maps reads at high edit distances, possibly containing indels, in a reasonable amount of time. TreQ does so at competitive running time compared with mrFAST, RazerS, SSAHA2 and BWA with customized parameters (BWA's default parameters are not competitive with respect to accuracy). The hybrid SOAP/TreQ approach, taking advantage of suffix-trie-based read mappers' efficiency on low edit distances and TreQ's sensitivity at higher edit distances, uses less time and maps more reads within all edit distances considered.

Although TreQ outperforms other read mappers on many mismatches or with large indels, its performance start to degrade gradually. As a result TreQ's specificity should drop at large edit distances. We have indirectly tested TreQ and other read mappers' specificity by combining the genome of human and chicken (a distant organism from human) and mapping the same one million real reads to this combined genome. We have found that BWA, BWA (customized), SOAP2, Novoalign, mrFAST and TreQ (within edit distance 18) map 87, 191, 65, 149, 189 and 187 reads, respectively, to the chicken genome. This experiment shows that TreQ's specificity is comparable with the other read mappers. For greater control over specificity, an optional maximum edit distance threshold *m* can be set in TreQ to discard any alignment with edit distance greater than *m* (default value 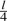, for read length *l*).

### 3.3 Memory requirements and multi-threading

The memory requirement for the cache-oblivious *kd*-tree and the *d*-dimensional vectors are 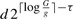 and 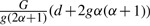, respectively. As a result, TreQ requires about 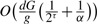 bytes of memory, given that 2*gα*(*α*+1)≤ *d*. Here, *G* is the genome size, *g* is the offset by which the genomic windows are shifted, Figure 1 (right), while creating *d*-dimensional *q*-gram vectors, 2*α* is the number of vectors for which we only store the changes from a nearby point and *τ* is the number of ignored lowest levels in the *kd*-tree. If we set *τ* = 4, *α* = 2 and *g* = 3, for *d* = 64 TreQ's memory requirement is around 40 GB (which is equivalent to using less than 1GB per core in a 48-core machine with the multi-threaded TreQ). Interestingly, these parameters have minor effects on accuracy and running time; for a detailed analysis, see Supplementary Figure S2. In addition, memory requirement can be further reduced by creating separate *kd*-trees for each chromosome and loading one *kd*-tree at a time in the memory.

**Fig. 3.**
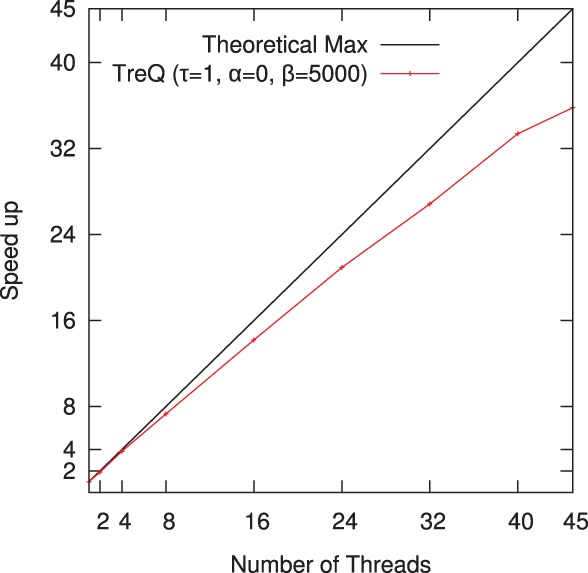
Speed up achieved by the multi-threaded version of TreQ on the task of mapping a randomly selected 0.1 million 101 bp single end reads from Yoruba African individual (NA18507).

We have developed a multi-threaded version of TreQ and tested its performance by mapping 0.1 million randomly selected Illumina single end reads (Yoruba African individual, NA18507) on a 48-core AMD Opteron 2.2 GHz server with 256 GB memory. The performance of TreQ scales very well in the number of threads, within 84% of the achievable maximum up to 40 cores (Fig. 3).

## 4 CONCLUSION

We address the problem of mapping NGS reads in an indel-tolerant manner by establishing geometric embedding as a promising paradigm, allowing identification of structural variants, including one or several indels of length 1–16 bp, from single read experiments: We map reads and genomic locations to trinucleotide frequency vectors, embedding them in ℝ^64^. The *L*_1_ distance between *q*-gram frequency vectors provides a lower bound for an edit distance with affine gap costs in which even long indels have small distance, improving the sensitivity of their detection. A probabilistic argument assuming reads, and genomes are generated from a Markov chain provides insight into the quality of the bound.

The problem of approximate matching is thus transformed into one of computing nearest neighbors using a spatial index. The decision for *kd*-trees was based on their favorable performance in many real-world scenarios (Kibriya and Frank, 2007) and the fact that they can be easily implemented in a cache-oblivious manner (Arge *et al.*, 2005), a major factor as memory accesses constitute the predominant bottleneck on modern CPU architectures.

Apart from the mapping of reads to the *q*-gram frequency vectors, our method has running times and memory requirements, which are about constant for the operations involving the index in the length of the reads. In practice, because of word sizes on computers, we expect this to hold for sequences of length 100–1000 bp.

Currently, SMP computers with many cores and 128 GB are the preferable platform for TreQ as the memory requirement is high, even when comparable with the state-of-the-art when measured in memory per core. Fortunately, SMP servers are approaching the prices of clusters with a comparable number of cores and main memory, which make the SMP platform the more versatile option at the same price point and memory usage a lesser issue. Also, the spatial index we used is a straight-forward *kd*-tree variant implemented in a cache-oblivious manner, which certainly can be improved upon.

We are currently investigating a parallel distributed index, which will allow the use of TreQ on clusters. Generally, we expect further improvements in running times, and consequently in accuracy, from an spatial index tailored specifically to the high-dimensional, integer coordinate problem setting, e.g. an adaptation of X-trees (Berchtold *et al.*, 1996) or through the use of locality-sensitive hashing (Paulevé *et al.*, 2010). Additional improvements in terms of both memory and running time can be made by using batch processing for queries.

The simplistic evaluation of putative matches using Levensthein distance will be replaced by a statistical, quality-score aware analysis following the lead of Stampy (Lunter and Goodson, 2011) and LAST (Frith *et al.*, 2010; Hamada *et al.*, 2011), which attribute their success to a large degree to the quality of their putative hit filtering. In our geometric embedding, quality scores can be used while searching for putative hits by using floating-point or fixed-point arithmetic and fractional count contributions for low-quality nucleotides.

The approximate matching tasks differ depending on whether a read has one exact match, few matches of small Hamming or Levenshtein distance, few matches of large Levenshtein distance with large indels or many matches of arbitrary distance. The resulting running times and accuracies depend heavily on the exact composition of the read set with respect to the types of matches. This implies that for data sets which are expected to contain both reads with indels and reads with mutations, a hybrid approach might be the most sensible option, as exemplified by our hybrid method. Traditional read mappers that are fast on low-mutation reads can be used to filter the unproblematic reads and then use geometric embedding to map the remaining ones in an indel-tolerant fashion.
